# Tumor suppressor prostaglandin D_2_

**DOI:** 10.18632/oncoscience.53

**Published:** 2014-06-13

**Authors:** Shingo Maeda, Tatsuro Nakamura, Takahisa Murata

**Affiliations:** Department of Animal Radiology, Graduate School of Agriculture and Life Sciences, The University of Tokyo, Tokyo

Colorectal cancer is the cause of death for some 600,000 people per year in the world and patient numbers are still increasing. Risk of colorectal cancer is greatly increased by inherited factors, lifestyle (smoking or alcohol consumption) and microbial infections causing bowel inflammation (colitis). The prolongation of colitis leads to tumorigenesis. The mechanism by which chronic colitis develops into colorectal cancer was thought to involve the production of a range of bioactive substances by immune cells invading intestinal tissue in response to inflammation. The produced bioactive substances then stimulate resident cells into abnormal growth. There would be a good chance of suppressing the emergence of colorectal cancer by preventing the inflammation.

The arachidonic acid-cyclooxygenase (COX) pathway generates a number of bioactive metabolites. These metabolites, also known as prostanoids, have been shown to be involved in a wide variety of inflammatory responses. Since epidemiologic studies demonstrated that the inhibition of COX by nonsteroidal anti-inflammatory drugs (NSAIDs) is protective against colorectal cancer, researchers have investigated the role of prostanoids in development and progression of colon cancer. Currently, there are several reports showing that major prostanoids prostaglandin E_2_ (PGE_2_) and thromboxane A_2_ (TXA_2_) promote colitis and colitis-associated colon cancer (CAC) in experimental animal models [1, 2]. Thus the usage of NSAIDs seems to be beneficial against colitis and CAC. However, its long-term usage causes serious side effect such as gastric ulcer suggesting each prostanoid possesses a pro- or an anti-inflammatory role and acts coordinately to govern pathophysiological responses of intestine. There are still urgent needs to investigate each role of prostanoid in colitis and CAC to develop a new therapeutic strategy with fewer side effects.

PGD_2_ is one prostanoid produced by activation of COX and PGD synthase. Mast cells, macrophages, and helper T (Th) 2-type lymphocytes are reported to express hematopoietic PGD synthase (H-PGDS) and potentially produce PGD_2_. PGD_2_ displays properties of both anti- and pro-inflammatory bioactivities through the G protein-coupled receptor DP and/or the chemoattractant receptor-homologous molecule expressed on Th2 cells (CRTH2). Previously, we found that mast cells release the abundant PGD_2_ and it acts as an anti-inflammatory and anti-angiogenic factor in the model of lung inflammation and implanted lung carcinoma [3, 4].

In our current publication, we have discovered that mast cell-derived PGD_2_ is a new bioactive substance inhibiting persistent colitis and subsequent colorectal cancer [5]. Systemic deficiency in H-PGDS aggravated colitis and accelerated tumor formation accompanied by increased TNF-α expression. Histologic analysis revealed that infiltrated mast cells strongly expressed H-PGDS in inflamed colons. Mast cell-specific H-PGDS deficiency aggravated colitis and accelerated tumorigenesis. Treatment with a DP receptor agonist alleviated colitis and suppressed tumorigenesis in association with decreased TNF-α expression. These observations suggest that mast cell-derived PGD_2_-DP signaling pathway inhibits prolonged colitis and subsequent tumor formation. Our findings provide fundamental information of the key role of PGD_2_ and mast cells in tumor immunity and a new therapeutic strategy against colon cancer.

Mast cells are well-known players to cause allergic inflammation. Of interest, these cells are also often found around tumor sites. Several growth factors and chemokines such as stem cell factor (SCF) released from the tumor microenvironment are likely to recruit mast cells. Recently, mast cells emerged as key players in tumor progression. Coussens et al. showed that mast cell deficiency exhibited the ablation of pre-neoplastic polyp development in the APC^min^ mice [6]. In contrast, Sinnamon et al. demonstrated that mast cells promote tumor apoptosis directly and/or indirectly through the recruitment of eosinophils [7]. Thus, the role of mast cells in tumorigenesis is still controversial. Indeed, pathological studies have been shown both a positive and a negative correlation between the mast cell number and prognosis in various human tumor types. Although we here identified PGD_2_ as a mast cell-derived anti-tumorigenic factor in murine CAC model, additional work will be required to clarify the role of mast cells in various types and stages of cancer.
